# Appendiceal Mucinous Neoplasms and Inflammatory Bowel Disease: Systematic Review of the Literature

**DOI:** 10.3390/jcm13010191

**Published:** 2023-12-29

**Authors:** Alessandro Michele Bonomi, Luca Ferrario, Alice Frontali, Piergiorgio Danelli, Francesco Colombo

**Affiliations:** 1 Department of General Surgery, Department of Biomedical and Clinical Sciences “L. Sacco”, University of Milan, ASST Fatebenefratelli Sacco, 20157 Milan, Italy; alessandro.bonomi@unimi.it (A.M.B.);; 2 Coloproctology and Inflammatory Bowel Disease Surgery Unit, IRCCS San Raffaele Scientific Institute, Via Olgettina 60, 20132 Milan, Italy

**Keywords:** appendiceal mucinous neoplasm, Crohn’s disease, ulcerative colitis, systematic review

## Abstract

There is no clear evidence on the prevalence and clinical presentation of appendiceal mucinous neoplasm (AMN) among patients with inflammatory bowel disease (IBD), so a systematic review was performed to investigate the diagnosis, management and treatment of AMN in these patients. PubMed, Medline, Scopus and the Cochrane Library were searched for articles published up to September 2023. Twenty-three studies reporting data about 34 AMN patients were included. UC patients had a median age of 52 years and a median length of disease of 10 years; CD patients had a median age of 40.5 years and a median length of disease of 5 years. A pre-operative diagnosis was achieved in 44% of patients. Most patients were symptomatic (82.6%) and showed moderate–severe disease activity (61%). Surgical procedures were performed: laparoscopic appendectomy, ileocecal resection, right hemicolectomy and colectomy/proctocolectomy. Of the patients, 73.5% were diagnosed with low-grade mucinous neoplasm (LAMN) and nine with adenocarcinoma. Synchronous colorectal dysplasia/carcinoma was present in 23.5% of patients. IBD patients with long-standing disease should be routinely screened, not only for colorectal cancer but also for AMN, during gastro-enterologic follow-up. Laparoscopic appendectomy of unruptured LAMN as well as right hemicolectomy of non-metastatic adenocarcinoma are safe procedures in IBD patients.

## 1. Introduction

Appendiceal mucinous neoplasms (AMNs), typically presenting as mucin-containing cystic masses, are rare lesions reported in less than 1% of appendectomy specimens [1,2]. The World Health Organization classifies the majority of noninvasive epithelial lesions as low-grade appendiceal mucinous neoplasms (LAMNs) [3]. Histologically, LAMNs are characterized by well-differentiated mucinous epithelial proliferations with low-grade cytologic atypia and any of the following: loss of muscularis mucosae, fibrosis of submucosa, pushing invasion and undulating or flattened epithelial growth. They can proliferate outside the appendix with a non-negligible risk of malignancy. Acellular or cellular extra-appendiceal mucin may be associated with LAMNs, although this is not mandatory. Under the term “LAMN”, the new terminology includes lesions that were described previously as mucoceles or mucinous cystadenomas, which are terms no longer in use. High-grade appendiceal neoplasms (HAMNs) share some histologic features with LAMNs but exhibit more aggressive cytologic atypia. Finally, mucinous adenocarcinomas are characterized by invasive glands containing high-grade cytologic atypia and extracellular mucin in >50% of the lesion. They can be defined as well, moderately or poorly differentiated; in addition, poorly differentiated tumors can be divided into neoplasms with or without signet ring cells (if containing <50% or >50%, respectively, of signet ring cells). Most patients are asymptomatic. When symptoms occur, they are commonly non-specific, prompting investigations for presumptive appendicitis or gynecologic etiologies. Rarely, they cause anemia/gastrointestinal bleeding, intestinal obstruction, intussusception, ureteric obstruction, volvules or cutaneous fistulae [4].

AMNs can develop extensions or can rupture over time. When this occurs, regardless of the degree of atypia, the patient is at risk of developing pseudomyxoma peritonei (PMP), a severe clinical syndrome characterized by progressive accumulation of a mucinous tumor throughout the peritoneal cavity, deeply worsening the prognosis [5].

On imaging studies, AMN appears as an appendix abnormally distended by mucin. Non-neoplastic etiologies (simple mucus retention cysts, for example) only account for about 20% of these lesions and usually measure less than 2 cm [5]. On the contrary, neoplastic ones tend to be larger at presentation [6]: cystic appendiceal dilatation with maximal diameter ≥15 mm on computerized tomography (CT) and/or ultrasound (US) have been reported to confidently diagnose AMN (CT, 71–83% sensitive and 88–92% specific [3]; US, 83% sensitive, 92% specific [7]).

However, in the majority of cases, pre-operative studies offer little help in identifying those with malignant potential, while up to 60% of appendiceal neoplasms are diagnosed either during surgical intervention or upon definitive histology [4]. For these reasons, surgery remains the cornerstone of treatment, and it should be an appendectomy or right hemicolectomy based on the extent of the disease [1].

Actually, there is no clear evidence on the prevalence and clinical presentation of AMN among patients with inflammatory bowel disease (IBD), but a well-documented higher risk of gastrointestinal malignant transformation [8] as well as growing evidence of appendiceal involvement [9,10] warrants particular consideration when dealing with mucin-containing appendix masses in these patients.

Given the scarcity of evidence, the aim of our manuscript is to carry out a systematic review of the literature attempting to define the magnitude of the issue and the best treatment approach for AMN in IBD patients.

## 2. Materials and Methods

### 2.1. Protocol Registration

The protocol was registered with the International Prospective Register of Systematic Reviews (PROSPERO ID, CRD42021237253). The study was reported according to the Preferred Reporting Items for Systematic Reviews and Meta-Analyses (PRISMA) 2020 statement [11].

### 2.2. Study Characteristics

All published studies regarding adult patients with histologically confirmed AMN affected by IBD—both Crohn’s disease (CD) and ulcerative colitis (UC)—subjected to surgical resection were included for review. Exclusion criteria were patients not affected by IBD, articles regarding editorials, conference abstracts, pre-clinical studies, previous reviews or book chapters and articles not in English. Given the scarcity of evidence, case reports were included.

### 2.3. Information Sources, Study Selection and Data Extraction

PubMed, Medline, Scopus and Cochrane Library databases were screened up to September 2023; search terms are reported in Appendix S1. Article screening was performed independently at the abstract level by two authors (LF, AB), excluding studies not meeting the inclusion criteria. The full texts of remaining studies were obtained and independently assessed by the mentioned authors. Disagreements were solved by third author (FC). Data from studies included were extracted onto a Libreoffice™ spreadsheet (Version 7.4). Qualitative assessment of studies was performed using the Joanna Briggs Institute Critical Appraisal Checklist for Case Reports [12] and the Newcastle–Ottawa quality assessment scale for case–control studies [13].

The following data were extracted: name(s) of the author(s), year of publication, type of study, age, male/female ratio, etiology (UC vs. CD), history of IBD diagnosis (years), presence of symptoms, pre-operative studies, pre-operative diagnosis, inflammatory disease activity, surgical approach (laparoscopic vs. open; urgent vs. elective), type of surgical procedure, lesion’s greatest dimension (cm), presence of synchronous colonic dysplasia/carcinoma, follow-up (months). Data were reported as mean (in case of normal distribution; otherwise as median) and proportions.

## 3. Results

**Figure 1:**
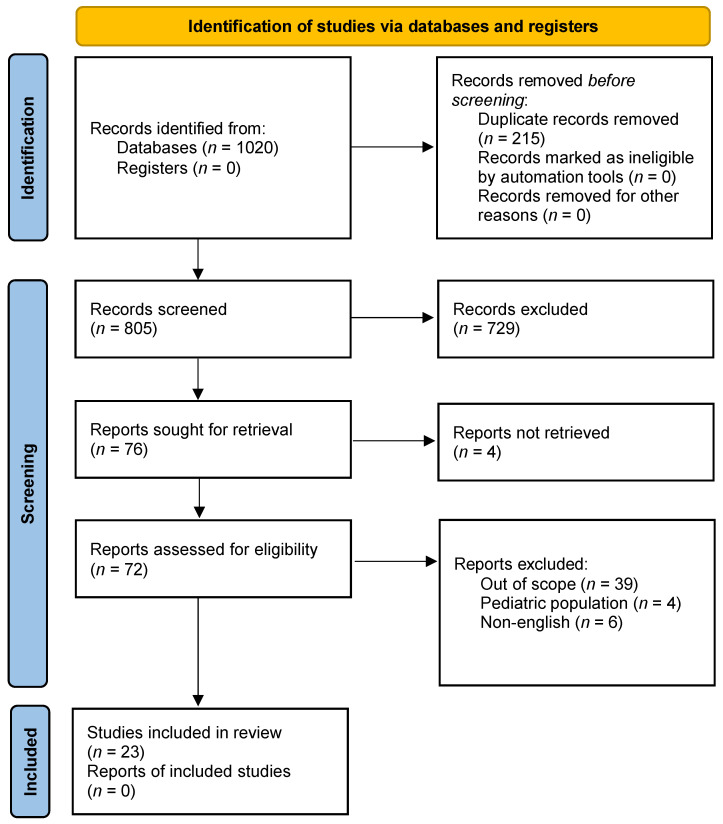
PRISMA flow-chart.

**Figure 2:**
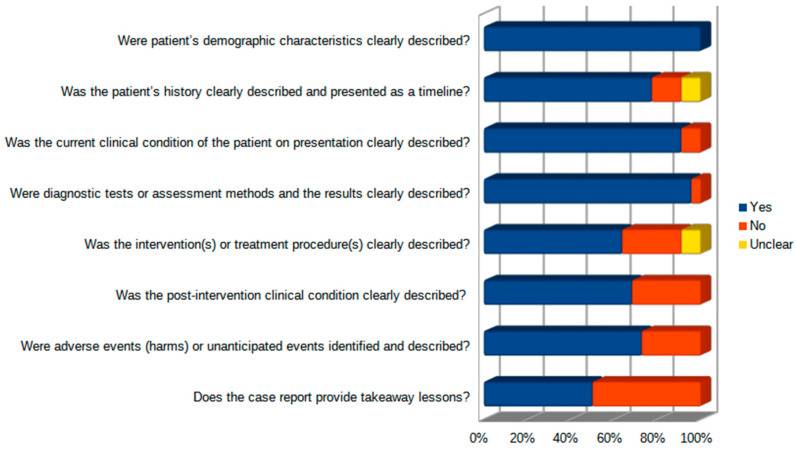
Joanna Briggs Institute Critical Appraisal Checklist for Case Reports results.

From the total of 1020 records identified (Figure 1), 805 were screened after duplicates were removed. Of these, 729 (90.6%) were excluded at the abstract level. Of the 76 reports remaining, 4 (5.3%) could not be retrieved, leaving 72 articles assessed for eligibility. After the full-text review, 49 records were excluded. Reasons for exclusion were the following: out of scope (*n* = 39); pediatric population (*n* = 4); non-English (*n* = 6). Overall, 23 studies met the inclusion criteria and reported data on 34 patients: 22 case reports and 1 retrospective case-control study (Newcastle–Ottawa scale: 6; Selection ***; Comparability 0; Exposure ***). Within the case reports, patient demographics, history and clinical condition were adequately described. On the other hand, intervention, post-intervention conditions, adverse events and takeaway lessons suffered an unclear description (Figure 2).

### 3.1. Patients’ Characteristics

Table 1 summarizes patients’ characteristics. 

Among the 34 patients, 26 had UC (76.5%), while 8 had CD (23.5%). The male/female ratio was 19/15, with a median age of 50 years (range 22–76). Patients with UC had a median age of 52 years (24–76); patients with CD had a median age of 40.5 years (22–68). 

A past history of IBD was reported in 18 studies; patients with UC had a median length of disease of 10 years (0–35). Patients with CD had a median length of disease of 5 years (0–10); 60% of patients with CD had AMNs as part of the presenting clinical symptoms of the new onset of their IBD. 

Clinical presentation was reported by 20 studies: 82.6% of patients were symptomatic, with right lower quadrant (RLQ) pain as the most frequent symptom (73.7%), and 17.4% were asymptomatic.

Disease activity was reported in 15 studies: 61% of patients had concomitant moderate–severe disease activity. Among patients with UC, seven had pancolitis and seven had proctitis–proctosigmoiditis. All CD patients showed ileocolic or cecal involvement. 

A pre-operative diagnosis was achieved in 15 patients: 11 of them were symptomatic (RLQ pain, diarrhea, bleeding) and CT was used in 12 patients. In the remaining patients, acute abdomen (RLQ pain mimicking acute appendicitis, UC not responsive to medical therapy and perforation) represented surgical indication; a diagnosis of AMN occurred either during surgical intervention or at a definitive histologic exam. 

Among 17 patients undergoing colonoscopy, 10 showed signs of peri-appendiceal involvement (mostly a protruding mass into the caecum). 

The median dimension of AMNs was 2 cm (0.4–14 cm); the median dimension of AMNs in symptomatic patients was 3.95 cm (2–14 cm). 

### 3.2. Surgical Management

Table 2 summarizes the surgical management, histology and follow-up.

Surgical procedures performed included: appendectomy (6), cecal resection (3), ileocecal resection (1), right hemicolectomy (10), colectomy/proctocolectomy (5). Most of the procedures were planned (17 elective), with only 8 emergency surgeries; in 9 cases timing was not reported. Only in 17 cases was a surgical approach reported, with 7 being laparoscopic and 10 open approaches.

Indications for extended resections other than appendectomy were the following: colic perforation (one), synchronous IBD disease requiring surgery (three), no clear margin on the base of the appendix (nine), synchronous dysplasia/colon cancer in UC (six).

Histology results reported 25 patients with LAMN (73.5%) and 9 with mucinous adenocarcinoma (26.5%); only in 1 case was a signet cell component reported. In UC patients, 77% had LAMN (20 of 26) against 62.5% in CD (5 of 8).

Synchronous colorectal dysplasia/carcinoma was present in eight patients (23.5%); six of them were diagnosed with LAMN; seven of them were affected by UC.

Follow-up data were reported by 12 studies: the median follow-up time was 9 months (1–120 months). One patient with positive margins after cecal resection underwent subsequent right hemicolectomy and was free of disease after 4 years of follow-up; one patient with CD and LAMN was lost to follow-up and developed pseudomyxoma peritonei 10 years later; one post-operative death was reported (an acute onset of CD complicated by signet ring adenocarcinoma of the appendix) and one patient developed a UC relapse soon, with a later right hemicolectomy requiring anti-TNF drugs.

## 4. Discussion

The absolute risk for the development of AMN among patients with IBD is not well defined; there is only one case–control retrospective study that investigated differences in the prevalence of AMN in IBD patients compared to the general population [14]: among 1203 colectomy specimens, LAMNs were found in 11 patients (0.9%), with 9 of them among 705 patients with IBD (1.3%) and 2 of them among 498 patients with other intestinal disorders (0.4%). Their results, however, did not reach statistical significance. However, the notion of a relationship between IBD and AMN stems from various physio-pathological mechanisms. 

Firstly, authors reported a high prevalence of chronic mucosal inflammation in the appendices of IBD patients: a review by Park highlighted that “Ulcerative appendicitis” can occur in up to 88% of colectomy specimens and up to 75% of endoscopic studies [9], while Crohn’s appendicitis occurs in 40–52% of colectomy specimens, especially those with extensive disease [10]. It has been suggested that appendiceal orifice inflammation may block luminal excretion, resulting in the occurrence of AMNs [15]. Among patients in our review, 58.8% showed peri-appendiceal involvement. 

Secondly, disease duration and histological inflammatory activity are both independent risk factors for the development of gastrointestinal dysplasia and carcinoma in patients with IBD, especially those affected by UC [8,16]. It is reasonable to hypothesize that long-standing and/or aggressive appendiceal inflammation related to IBD should predispose patients to AMN.

This hypothesis finds support in our data: the median age of UC patients with AMN was 52 years, the mean length of disease was 10 years and 61% of all patients showed moderate or severe disease activity. Interestingly, 60% of AMNs in CD patients were part of the presenting clinical picture of previously unknown CD and were associated with ileocolic disease with surgical indication. Probably, aggressive disease biology plays an adjunctive role in tumorigenesis. Taking into account the limitations deriving from the limited number and quality of evidence, given these indications, we believe it is reasonable to consider the possibility of a direct connection between IBD and AMN.

The clinical presentation of AMN in IBD patients seems to be similar to that of the general population. A recent systematic review of 276 AMN cases by Morano et al. [4] highlighted right abdominal pain as the most frequent symptom (60.8% vs. 73.7%) and a similar proportion of asymptomatic patients (22.8% vs. 17.4%). Nausea/emesis, weight loss, rectal bleeding/bloody diarrhea, palpable abdominal mass, abdominal bloating and discomfort are amongst the reported non-specific signs and symptoms associated with the presence of AMN. 

AMN should be considered when encountering symptomatic appendiceal masses greater than 2 cm [7,17]. That holds true in particular for acute abdomen, as some authors state that appendiceal adenocarcinoma most commonly presents with acute appendicitis [17,18]. Accordingly, in our series, the AMNs’ mean diameter in symptomatic patients was 3.95 cm (2–14 cm); two patients presented with symptoms mimicking acute appendicitis and were all diagnosed with appendiceal adenocarcinoma.

Nonetheless, prompt diagnosis seems to be more challenging in IBD patients: we found a higher rate of incidentally discovered AMN in IBD patients compared to Morano’s review (55.9% and 38.4%, respectively) [4]. Probably, overlapping underlying disease relapses might explain this finding.

US and RM were not routinely performed in pre-operative studies. However, given their extensive use in investigating elective cases and acute exacerbations of IBD, awareness of this rare disorder is required to avoid misinterpretation of AMN. 

Ultrasonography can show an elongated hypoechoic mass due to increased dilatation of the proximal portion of the appendix. Features highly suggestive of an AMN are internal concentric echogenic layers giving the appendix an onion skin appearance and acoustic shadowing due to dystrophic mural calcifications (though present in less than 50% of cases) [6,7].

With magnetic resonance imaging (MRI), an AMN most frequently demonstrates characteristics of a simple fluid lesion, although signal intensity varies depending on the specific protein content. Appendiceal wall calcifications and intraluminal gas are more difficult to appreciate with MRI than with CT [6].

Once a presumptive diagnosis is suggested, CT should be routinely used in these neoplasms (it was reported in 85.7% of our cases), as it helps to rule out or confirm the diagnosis and allows precise observation of the relation between the lesion and the neighboring organs. 

Typical CT features include a well-demarcated round or tubular structure with homogeneous near-water attenuation and an enhancing wall in the expected site of the appendix. Mural nodularity and irregular wall thickening are features that have been associated with adenocarcinoma, while maximal wall thickness, the presence of internal septa, wall calcifications, peri-appendiceal fat stranding and/or intraperitoneal free fluid are not helpful in differentiating malignant from benign disease [5,6].

Colonoscopy is also suggested, especially in IBD patients [1]. Endoscopically, AMN is suggested either when a distended appendiceal orifice is observed, or in the presence of caecum indentation/elevation or yellowish mucous discharge coming from the appendiceal orifice [9].

Wong et al. report EUS as a useful imaging modality to distinguish intramural from extracolonic lesions [19]. Interestingly, 58,8% of patients undergoing colonoscopy showed appendiceal involvement, including UC patients that had active disease limited to the sigmoid colon and rectum. 

Surgery remains the standard of treatment [1]. However, the optimal surgical approach and the appropriate extent of resection remain controversial. The most important factors determining the appropriate extent of resection are the safety margin of the AMN and the preserved anatomy of its base [20]. As underlined in the latest guidelines, appendectomy is recommended for simple AMN with an intact appendiceal base, either diagnosed pre-operatively or if a grossly abnormal appendix is encountered during an unrelated abdominal operation [1]. Careful dissection is imperative throughout the intervention to avoid rupture and spillage of cells in the peritoneal cavity. Partial cecal resection is required when the base of the AMN is broad and protrudes into the cecal wall. Ileocecal resection or right hemicolectomy is recommended if an adequate resection margin cannot be secured and if malignancy is strongly suspected. 

In our series, 23.5% of patients had synchronous colorectal dysplasia/carcinoma; notably, the American Society of Colon and Rectal Surgeons (ASCRS) guidelines strongly recommend routine use of colonoscopies in patients with AMN [1]. As a consequence, we feel it is reasonable to state that surgical strategy in IBD cases should take into account colectomy/proctocolectomy as a therapeutic option for synchronous disease activity requiring surgical treatment as well as synchronous dysplasia/carcinoma. 

As reported by Kim et al. [20], laparoscopy can be safely used. In their series of 58 patients affected by AMN who underwent laparoscopic surgical intervention, they report similar operative times, higher rates of appendectomy, shorter lengths of hospital stays and similar rates of appendiceal rupture compared to controls who underwent open surgery. Their univariate analysis identified leukocytosis (white blood cell count > 10,000/μL) as the only risk factor for intra-operative appendiceal perforation, while an AMN diameter greater than 2 cm was not associated with a significantly higher risk of rupture. ASCRS guidelines suggest that if a LAMN cannot be safely resected laparoscopically, conversion to an open operation is recommended.

The following strategy depends on definitive histologic reports. According to the latest ASCRS guidelines [1], patients with LAMN/HAMN with negative margins and no evidence of perforation or peritoneal involvement are safely treated with appendectomy. In patients with evidence of HAMN, care should be taken to exclude the presence of associated invasive adenocarcinomas, including comprehensive histologic evaluation of the entire surgical specimen. 

Patients with non-metastatic adenocarcinoma of the appendix should undergo right hemicolectomy. 

In our series, patients with adenocarcinoma were treated either with right hemicolectomy or with proctocolectomy (in UC patients). Only Takeda reported the presence of an in situ carcinoma in the appendiceal orifice treated with appendectomy, with five months of follow-up negative for relapse [21].

Regardless of surgical strategy, a thorough abdominal exploration should be carried out to exclude the presence of mucinous peritoneal implants and/or mucinous ascites, especially in case of AMN rupture. In our series, iatrogenic or spontaneous rupture of AMN was not reported. However, in the setting of peritoneal spread, individualized decisions regarding cytoreductive surgery with or without intraperitoneal hyperthermic chemoperfusion (HIPEC) should be undertaken by a multidisciplinary team, preferably at experienced centers. 

Long-term follow-up is warranted but remains complex, especially in IBD patients. Recent studies reporting adequate follow-up (ranging from 2.6 to 4.8 years) through a combination of CT, diagnostic laparoscopy and tumor markers described a peritoneal recurrence rate ranging from 4.9% to 52% [22,23,24,25]. While all of these authors agree that radically resected LAMN carries a low recurrence risk, we still lack tools to identify the subset of patients at higher risk for peritoneal recurrence. Elevated tumor markers’ levels (CEA, CA 19.9, CA 125) at primary surgery and/or follow-up, spontaneous or iatrogenic rupture, positive resection margins and mucin in the appendiceal submucosa wall or peri-appendiceal tissue are features that should prompt careful follow-up [26]. In addition, there is evidence that patients with appendiceal neoplasms are at increased risk of synchronous colonic lesions compared with the general population: in a population-based study from the Netherlands from 1995 to 2005 that included 1482 patients with an appendiceal epithelial neoplasm, 10.5% had an incidental colonic adenocarcinoma [27].

Orta et al. [14] stated that AMNs could be considered a neoplastic complication of inflammatory diseases: in their study, among 705 colectomy specimens from patients with IBD, the subset of specimens with colonic dysplasia/cancer showed a significantly higher prevalence of LAMN (5.8%) compared to its prevalence among uncomplicated IBD (0.8%) and controls (0.9%). Therefore, adequate planning of appropriate endoscopic follow-up is warranted and surgical strategy should take into account these assumptions (e.g., indication to proctocolectomy).

## 5. Conclusions

AMNs remain a rare clinical entity in IBD patients: to this date, current evidence relies on 22 [18,19,21,28,29,30,31,32,33,34,35,36,37,38,39,40,41,42,43,44,45,46] case reports and only 1 retrospective case–control study [14]. To our knowledge, this is the very first review of cases of AMN in IBD patients.

Even if available evidence relies on very low-quality and scarce data, which prevents us from drawing rigorous conclusions, we feel it is reasonable to state that IBD patients with long-standing disease should be routinely screened not only for colorectal cancer but also for AMN during gastro-enterologic follow-up, meaning that screening colonoscopies and imaging studies should investigate and report adequate information about the appendix. This would not have an excessive economic impact: abdominal ultrasound and colonoscopy are examinations already provided for in the regular follow-up of IBD patients. Laparoscopic appendectomies of unruptured LAMNs/HAMNs as well as right hemicolectomies of non-metastatic adenocarcinomas are safe procedures and associated with a low risk of recurrence; extensive resections should be discussed in a multidisciplinary setting in IBD tertiary care centers. Long-term follow-up is recommended. 

## Figures and Tables

**Table 1:** Patients’ characteristics.

Author	Year of Publication	Years of Enrollment	Type of Study	No. of Surgically Treated Patients	Age	Male/Female	Etiology	Associated Disease Activity	Description of Disease Activity
Moniakis	2021		Case report	1	65	F	CD	Yes	Remission
Davey	2020		Case report	1	42	M	UC	Yes	Proctitis
Ghosh	2010		Case report	1	55	F	UC	NR	NR
Lakatos	2005		Case report	1	60	M	UC	Yes	Proctosigmoiditis
Lyda	1998		Case report	1	48	M	UC	NR	NR
Nehme	2019		Case report	1	42	M	UC	Yes	Pancolitis
Noaki	2009		Case report	1	33	F	UC	No	Proctosigmoiditis
Orta	2009	2002–2006	Retrospective	9	46.2 (mean)	6M/3F	6 UC 3 CD	NR	4 pancolitis (UC); 2 left colitis (UC); 3 ileocolic (CD)
Kuester	2008		Case report	1	36	M	UC	Yes	Pancolitis
Shen	2014		Case report	1	68	M	UC	No	Remission
Sonwalkar	2002		Case report	1	68	F	CD	Yes	Caecum
Takeda	2008		Case report	1	42	M	UC	No	NR
Tonolini	2015		Case report	1	71	M	UC	Yes	Pancolitis
Villanueva Saenz	2006		Case report	1	60	F	UC	Yes	Left colitis
Vukovic	2018		Case report	1	22	M	CD	Yes	Ileocolic
Zannoni	1997		Case report	1	69	F	UC	No	Remission
Wong	2012		Case report	2	48 (mean)	2F	2 UC	No/Yes	Remission/Pancolitis
Simsek	2019		Case report	1	22	F	CD	Yes	Ileocolic
Abdulghaffar	2022		Case report	1	48	F	UC	Yes	Proctosigmoiditis
Almogy	2001		Case report	1	38	M	CD	Yes	Ileocolic
Fukumoto	2021		Case report	1	52	M	UC	Yes	Left colitis
Klag	2016		Case report	1	63	M	UC	Yes	Remission
Fakheri	2023		Case report	3	55.7 (mean)	2F; 1M	3 UC	Yes	2 pancolitis; 1 left colitis

M = male; F = female; CD = Crohn’s disease; UC = ulcerative colitis; NR = not reported.

**Table 2:** Surgical management, intra-operative findings, histology.

Author	Publication Year	Surgical Approach	Timing	Surgical Procedure	Reason for Extended Resection (Other than Appendectomy)	Histologic Report	Pathological Stage + Extra-Appendiceal Mucin	Synchronous Colon Dysplasia/Carcinoma
Moniakis	2021	Laparotomy	Urgent	right hemicolectomy	NR	LAMN	No mucin	0
Davey	2020	Open	Urgent	right hemicolectomy	no clear margin	LAMN	TisN0, no mucin	0
Ghosh	2010	Laparoscopic	Elective	right hemicolectomy	no clear margin	LAMN	No mucin	0
Lakatos	2005	Open	Elective	cecal resection	no clear margin	LAMN	No mucin	0
Lyda	1998	Open	Elective	proctocolectomy	dysplasia in UC	LAMN	No mucin	1
Nehme	2019	NR	Urgent	proctocolectomy with ileostomy	dysplasia in UC	Mucinous adenocarcinoma	TNM NR, no mucin	1
Noaki	2009	Open	Elective	cecal resection	no clear margin	LAMN	No mucin	1
Orta	2009	NR	NR	colectomy	NR	LAMN	NR	4 UC
Kuester	2008	NR	Elective	proctocolectomy with ileostomy	dysplasia in UC	LAMN	No mucin	1
Shen	2014	Laparoscopic	Urgent	right hemicolectomy	no clear margin	Mucinous adenocarcinoma	T2, N not reported No mucin	0
Sonwalkar	2002	Open	Elective	right hemicolectomy	colon cancer (caecum)	Mucinous adenocarcinoma	T4N1M0, no mucin	1
Takeda	2008	NR	Urgent	appendectomy	appendectomy	Mucinous adenocarcinoma	TNM NR, no mucin	0
Tonolini	2015	Laparoscopic	Elective	appendectomy	appendectomy	LAMN	No mucin	0
Villanueva Saenz	2006	NR	Elective	proctocolectomy with ileostomy	severe pancolitis	Mucinous adenocarcinoma	TNM NR, no mucin	0
Vukovic	2018	NR	Urgent	right hemicolectomy	IBD relapse	Mucinous adenocarcinoma + signet ring cells	TNM NR, no mucin	0
Zannoni	1997	Open	Elective	right hemicolectomy	no clear margin	Mucinous adenocarcinoma	TNM NR, no mucin	0
Wong	2012	Laparoscopic	Elective	appendectomy	appendectomy	LAMN	No mucin	0
Simsek	2019	Open	Elective	ileocecal resection	IBD relapse	LAMN	PMP, TNM NR	0
Abdulghaffar	2022	Open	Elective	appendectomy	appendectomy	LAMN	No mucin	0
Almogy	2001	Open	Urgent	subtotal colectomy	colon perforation	Mucinous adenocarcinoma	TNM NR, no mucin	0
Fukumoto	2021	Laparoscopic	Urgent	right hemicolectomy	suspected cancer of right colon	Mucinous adenocarcinoma	T4bN1aM0, no mucin	0
Klag	2016	Laparoscopic	Elective	cecal resection	no clear margin	LAMN	No mucin	0
Fakheri	2023	NR	Elective	2 right hemicolectomies + appendectomy	no clear margin	3 LAMN	No mucin	0

LAMN = low-grade appendiceal mucinous neoplasm; UC = ulcerative colitis; NR = not reported; PMP = pseudomyxoma peritonei.

## Data Availability

The data that support the findings in this study are available from the corresponding author upon reasonable request.

## Supplementary Materials

The following supporting information can be downloaded at: https://www.mdpi.com/article/10.3390/jcm13010191/s1, Appendix S1: Search strategy.

## References

[1] Glasgow S.C., Gaertner W., Stewart D., Davids J., Alavi K., Paquette I.M., Steele S.R., Feingold D.L. (2019). The American Society of Colon and Rectal Surgeons, Clinical Practice Guidelines for the Management of Appendiceal Neoplasms. Dis. Colon Rectum.

[2] Stopenski S.J., Grigorian A., Carmichael J., Mills S., Brady M., Dolich M., Kuza C.M., Nguyen N.T., Nahmias J. (2021). Risk Factors for Appendiceal Cancer after Appendectomy. Am. Surg..

[3] Carr N.J., Cecil T.D., Mohamed F., Sobin L.H., Sugarbaker P.H., González-Moreno S., Taflampas P., Chapman S., Moran B.J. (2016). A Consensus for Classification and Pathologic Reporting of Pseudomyxoma Peritonei and Associated Appendiceal Neoplasia: The Results of the Peritoneal Surface Oncology Group International (PSOGI) Modified Delphi Process. Am. J. Surg. Pathol..

[4] Morano W.F., Gleeson E.M., Sullivan S.H., Padmanaban V., Mapow B.L., Shewokis P.A., Esquivel J., Bowne W.B. (2018). Clinicopathological Features and Management of Appendiceal Mucoceles: A Systematic Review. Am. Surg..

[5] Bartlett D.J., Thacker P.G., Grotz T.E., Graham R.P., Fletcher J.G., VanBuren W.M., Iyer V.R., Fidler J.L., Menias C.O., Wasif N. (2019). Mucinous Appendiceal Neoplasms: Classification, Imaging, and HIPEC. Abdom. Radiol..

[6] Leonards L.M., Pahwa A., Patel M.K., Petersen J., Nguyen M.J., Jude C.M. (2017). Neoplasms of the Appendix: Pictorial Review with Clinical and Pathologic Correlation. Radiographics.

[7] Lien W.-C., Huang S.-P., Chi C.-L., Liu K.-L., Lin M.-T., Lai T.-I., Liu Y.-P., Wang H.-P. (2006). Appendiceal Outer Diameter as an Indicator for Differentiating Appendiceal Mucocele from Appendicitis. Am. J. Emerg. Med..

[8] Axelrad J.E., Lichtiger S., Yajnik V. (2016). Inflammatory Bowel Disease and Cancer: The Role of Inflammation, Immunosuppression, and Cancer Treatment. World J. Gastroenterol..

[9] Park S.H., Loftus E.V., Yang S.-K. (2014). Appendiceal Skip Inflammation and Ulcerative Colitis. Dig. Dis. Sci..

[10] Stangl P.C., Herbst F., Birner P., Oberhuber G. (2002). Crohn’s Disease of the Appendix. Virchows Arch..

[11] Page M.J., McKenzie J.E., Bossuyt P.M., Boutron I., Hoffmann T.C., Mulrow C.D., Shamseer L., Tetzlaff J.M., Akl E.A., Brennan S.E. (2021). The PRISMA 2020 Statement: An Updated Guideline for Reporting Systematic Reviews. Int. J. Surg..

[12] Aromataris E., Munn Z., Aromataris E., Munn Z. (2020). JBI Manual for Evidence Synthesis.

[13] Wells G., Shea B., O’Connell D., Peterson J.E., Welch V., Losos M., Tugwell P. (2000). The Newcastle–Ottawa Scale (NOS) for Assessing the Quality of Non-Randomized Studies in Meta-Analysis.

[14] Orta L., Trindade A.J., Luo J., Harpaz N. (2009). Appendiceal Mucinous Cystadenoma Is a Neoplastic Complication Of IBD: Case-Control Study of Primary Appendiceal Neoplasms. Inflamm. Bowel Dis..

[15] Matsushita M., Tanaka T., Omiya M., Okazaki K. (2010). Significant Association of Appendiceal Neoplasms and Ulcerative Colitis Rather than Crohn’s Disease. Inflamm. Bowel Dis..

[16] Uchino M., Ikeuchi H., Hata K., Minagawa T., Horio Y., Kuwahara R., Nakamura S., Watanabe K., Saruta M., Fujii T. (2021). Intestinal Cancer in Patients with Crohn’s Disease: A Systematic Review and Meta-Analysis. J. Gastroenterol. Hepatol..

[17] Teixeira F.J.R., do Couto Netto S.D., Akaishi E.H., Utiyama E.M., Menegozzo C.A.M., Rocha M.C. (2017). Acute Appendicitis, Inflammatory Appendiceal Mass and the Risk of a Hidden Malignant Tumor: A Systematic Review of the Literature. World J. Emerg. Surg..

[18] Nehme F., Schneider A., Hamid F. (2019). Appendiceal Adenocarcinoma Associated with Ulcerative Colitis. ACG Case Rep. J..

[19] Wong U., Darwin P. (2012). Appendiceal Mucocele Diagnosed in Patients with Inflammatory Bowel Disease Using Endoscopic Ultrasound. Case Rep. Med..

[20] Kim T.K., Park J.H., Kim J.Y., Kim B.C., Kang B.M., Min S.K., Kim J.W. (2018). Safety and Feasibility of Laparoscopic Surgery for Appendiceal Mucocele: A Multicenter Study. Surg. Endosc..

[21] Takeda Y., Nakase H., Mikami S., Inoue T., Satou S., Sakai Y., Chiba T. (2008). Possible Link between Ulcerative Colitis and in Situ Adenocarcinoma of an Appendiceal Mucocele: Importance of Inflammation in the Appendiceal Orifice Related to UC. Inflamm. Bowel Dis..

[22] Guaglio M., Sinukumar S., Kusamura S., Milione M., Pietrantonio F., Battaglia L., Guadagni S., Baratti D., Deraco M. (2018). Correction to: Clinical Surveillance After Macroscopically Complete Surgery for Low-Grade Appendiceal Mucinous Neoplasms (LAMN) with or Without Limited Peritoneal Spread: Long-Term Results in a Prospective Series. Ann. Surg. Oncol..

[23] Foster J.M., Sleightholm R.L., Wahlmeier S., Loggie B., Sharma P., Patel A. (2016). Early Identification of DPAM in At-Risk Low-Grade Appendiceal Mucinous Neoplasm Patients: A New Approach to Surveillance for Peritoneal Metastasis. World J. Surg. Oncol..

[24] Fournier K., Rafeeq S., Taggart M., Kanaby P., Ning J., Chen H.-C., Overman M., Raghav K., Eng C., Mansfield P. (2017). Low-Grade Appendiceal Mucinous Neoplasm of Uncertain Malignant Potential (LAMN-UMP): Prognostic Factors and Implications for Treatment and Follow-Up. Ann. Surg. Oncol..

[25] Honoré C., Caruso F., Dartigues P., Benhaim L., Chirica M., Goéré D., Elias D. (2015). Strategies for Preventing Pseudomyxoma Peritonei After Resection of a Mucinous Neoplasm of the Appendix. Anticancer Res..

[26] McDonald J.R., O’Dwyer S.T., Rout S., Chakrabarty B., Sikand K., Fulford P.E., Wilson M.S., Renehan A.G. (2012). Classification of and Cytoreductive Surgery for Low-Grade Appendiceal Mucinous Neoplasms. Br. J. Surg..

[27] Smeenk R.M., van Velthuysen M.L.F., Verwaal V.J., Zoetmulder F. (2008). Appendiceal Neoplasms and Pseudomyxoma Peritonei: A Population Based Study. Eur. J. Surg. Oncol..

[28] Moniakis A.A., Flamourakis M.E., Gkionis I.G., Giakoumakis M.I., Tsagkataki E.S., Kazamias G.M., Spiridakis K.G., Christodoulakis M.S. (2021). Ileocolic Intussusception in a Woman: A Case Report and Literature Review. Am. J. Case Rep..

[29] Davey M.G., Conlon E.T., Forde G., Byrnes V.M., Carroll P.A. (2020). Adult Intussusception Secondary to an Appendiceal Tumour in a Patient with Ulcerative Colitis: A Case Report. Surg. Case Rep..

[30] Ghosh T., Chalmers A., Verbeke C., Saunders R., Everett S. (2010). Appendiceal Mucocele in Ulcerative Colitis. Endoscopy.

[31] Lakatos P.L., Gyori G., Halasz J., Fuszek P., Papp J., Jaray B., Lukovich P., Lakatos L. (2005). Mucocele of the Appendix: An Unusual Cause of Lower Abdominal Pain in a Patient with Ulcerative Colitis-. A Case Report and Review of Literature. World J. Gastroenterol. WJG.

[32] Lyda M.H., Noffsinger A., Belli J., Fischer J., Fenoglio-Preiser C.M. (1998). Multifocal Neoplasia Involving the Colon and Appendix in Ulcerative Colitis: Pathological and Molecular Features. Gastroenterology.

[33] Noaki R., Kawahara H., Watanabe K., Kobayashi S., Uchiyama K., Yanaga K. (2009). Appendiceal Mucocele Detected under Treatment of Ulcerative Colitis. Case Rep. Gastroenterol..

[34] Kuester D., Dalicho S., Mönkemüller K., Benedix F., Lippert H., Guenther T., Roessner A., Meyer F. (2008). Synchronous Multifocal Colorectal Carcinoma in a Patient with Delayed Diagnosis of Ulcerative Pancolitis. Pathol. Res. Pract..

[35] Shen H., Lipka S., Katz S. (2014). Appendiceal Adenocarcinoma in a Patient with Chronic Ulcerative Colitis: What Is the Appropriate Surgical Procedure?. J. Crohns Colitis.

[36] Sonwalkar S.A., Denyer M.E., Verbeke C.S., Guillou P.J. (2002). Cancer of Appendix as a Presenting Feature of Crohn’s Disease. Eur. J. Gastroenterol. Hepatol..

[37] Tonolini M. (2015). Appendiceal Mucocele in Ulcerative Colitis: A Rare Association and a Crucial Preoperative Diagnosis. J. Gastrointest. Liver Dis..

[38] Pérez-Aguirre J., Villanueva Saenz E., Belmonte M.C., Martínez P.H.M., Márquez R.M.L., Carranza R.J.M. (2006). Appendix Adenocarcinoma Associated with Ulcerative Colitis: A Case Report and Literature Review. Tech. Coloproctol..

[39] Vukovic J., Vrebalov Cindro P., Tomic S., Tonkic A. (2018). Signet Ring Carcinoma of the Appendix Presenting as Crohn’s Disease in a Young Male. Case Rep. Gastroenterol..

[40] Zannoni U., Masci C., Bazzocchi R., Gandolfo F., Pezzi A., Alampi G., Biasco G. (1997). Cancer of the Appendix in Long-Standing Ulcerative Colitis: A Case Report. Tumori J..

[41] Simsek M., Linn A.J., de Boer N.K.H. (2019). Pseudomyxoma Peritonei of the Appendix after Ileocecal Resection: Expect the Unexpected. Dig. Liver Dis..

[42] Abdulghaffar S., Badrawi N., Khairi T.E., Keloth T.R., Businge P.E. (2022). Giant Appendiceal Mucocele as a First Manifestation in a Patient with Silent Ulcerative Colitis: A Case Report. Radiol. Case Rep..

[43] Almogy G., Fellig Y., Paz K., Durst A., Eid A. (2001). Adenocarcinoma of the Appendix Associated with Long-Standing Crohns Disease. Int. J. Color. Dis..

[44] Fukumoto Y., Kobayashi Y., Takemura S., Maeda K., Nakamura F., Inatomi O., Andoh A., Ban H. (2021). A Case of Appendix Adenocarcinoma Associated with Ulcerative Colitis. Clin. Case Rep..

[45] Klag T., Wehkamp J., Bösmüller H., Falch C., Johannink J., Malek N.P., Kirschniak A., Goetz M. (2017). Low-Grade Appendiceal Mucinous Neoplasm (LAMN)—3-Year Endoscopic Follow-up Underlines Benign Course of LAMN Type 1. Z. Gastroenterol..

[46] Fakheri H., Bari Z., Yaghoobi M., Rabiee P. (2023). Concomitant Occurrence of Appendiceal Mucocele and Ulcerative Colitis: Case Reports. Caspian J. Intern. Med..

