# Supplementary material for: Appendiceal Mucinous Neoplasms and Inflammatory Bowel Disease: Systematic Review of the Literature
Source: J Clin Med. 2023 Dec 29;13(1):191. doi: 10.3390/jcm13010191 (PMC10780220; doi:10.3390/jcm13010191)
Supplement: Supplementary file 1 [file jcm-13-00191-s001.zip › jcm-2785885-supplementary.pdf]

Appendix S1: Search strategy:

1) Appendix OR appendectomy OR appendiceal neoplasm OR mucocele OR appendiceal cancer OR appendiceal cystadenoma AND Crohn's disease
2) Appendix OR appendectomy OR appendiceal neoplasm OR mucocele OR appendiceal cancer OR appendiceal cystadenoma AND Ulcerative Colitis
